# The Curious Case of Cholangiocarcinoma: Opportunities for Environmental Health Scientists to Learn about a Complex Disease

**DOI:** 10.1155/2018/2606973

**Published:** 2018-08-09

**Authors:** William A. Suk, Vajarabhongsa Bhudhisawasdi, Mathuros Ruchirawat

**Affiliations:** ^1^Superfund Research Program, National Institute of Environmental Health Sciences, Research Triangle Park, Durham, NC 27709, USA; ^2^Surgery, Khon Kaen University, Changwat Khon Kaen, Khon Kaen 40002, Thailand; ^3^Research and Academic Affairs, Chulabhorn Research Institute, Bangkok 10210, Thailand

## Abstract

Deaths from complex, noncommunicable diseases such as cancer are predicted to continue to increase worldwide, with low- and middle-income countries bearing the brunt of the burden. This problem requires a concentrated global effort to avoid devastating losses of life as well as economic losses. Cholangiocarcinoma (CCA) is a readily studied model of complex, noncommunicable disease, but it receives little attention outside of the scientific community in Southeast Asia. Here, we bring attention to the opportunity to study CCA as a model to understand the role of multiple, complex factors in cancer. These factors include allostatic load, individual genetic susceptibility, and environmental exposures such as chemicals, diet, socioeconomic factors, and psychosocial stress. The study of CCA offers a unique opportunity to make novel observations that could advance progress in prevention and intervention approaches for prevalent diseases that involve complex, multifactorial interactions.

## 1. Introduction

Deaths from cancer and other noncommunicable diseases are expected to continue to rise, especially in low- and middle-income countries (LMICs) [1, 2]. The surge in cancer mortality is predicted to be especially dramatic in Southeast Asia, Southern China, and India, which face poverty, limited health care access, and other environmental conditions that intensify this burden [3]. This predicted burden on LMICs and the global increase in cancer, including in developed nations like the United States and the United Kingdom [4, 5], will cause catastrophic losses of life, in addition to economic devastation [3].

Complex diseases caused in part by environmental exposures are emerging as an increasing threat worldwide [1]. Our understanding of the rise in disease is complicated by the fact that human populations are incredibly diverse in their genes, lifestyles, daily environmental exposures, and other stressors, all of which can play a role in the multifactorial etiology of health and disease [6]. Disentangling the totality of exposures throughout the life-course and differences in the health impacts among individuals and populations is a challenge.

Studying diseases that are rare in the human population may offer a pathway to understanding many complex noncommunicable diseases. Because the affected populations are small and relatively well defined, it becomes easier to explore the environmental factors that may contribute to the disease. For example, researchers studying rare cancers have uncovered insights into diagnosing cancer, how cancer develops [7], gene mutations not previously implicated in cancer [8], and novel genes that can be used in diagnosis or classification of cancers [7]. Similarly, studying the interaction between aflatoxin and hepatitis B virus leading to liver cancer led to increased understanding of human environmental exposure levels, exposure assessment methods, metabolism of carcinogens and other toxicants, and mechanisms of carcinogenesis and other disease processes [9]. Thus, investigating rare diseases, including the underlying cellular mechanisms or genes involved, produces findings that can be translated to other more common diseases.

In this paper, we discuss some of the multiple, complex factors that may contribute to the rare bile duct cancer, cholangiocarcinoma (CCA), including diet and environmental exposures, genetic variations in susceptibility, coinfections and comorbidities, and socioeconomic and psychosocial factors. We illustrate how CCA is a real-world example of the effects of the exposome—the measure of all the endogenous and exogenous environmental exposures in an individual's lifetime and individual biological responses to these exposures [10]—and argue that understanding the full picture of CCA promises to improve treatment and prevention of this disease, and help disentangle the many complex factors involved in other noncommunicable diseases.

## 2. Cholangiocarcinoma as a Model Disease

Though rare in most of the world, CCA incidence and mortality has increased in recent decades in many countries including LMICs, the United Kingdom, and United States [11–13]. However, it has received little attention and has not been well studied outside of the scientific community in Southeast Asia. To understand this increase in deaths and the burden that CCA presents, it is important to understand all the factors that contribute to the disease.

### 2.1. The Winding Pathway to CCA

In Southeast Asia, CCA is often found in people who consume raw or undercooked fish or sea plants that are infected with one of two different types of parasitic liver flukes. Indeed, both species of liver flukes that most commonly infect humans (*Opisthorchis viverrini* and *Clonorchis sinensis*) have been declared Group 1 carcinogens by the World Health Organization and the International Agency for Research on Cancer [14, 15]. Although CCA has been known to be a complex disease with multiple risk factors since 1942 [16], in much of the limited study to date, investigations have primarily explored the obvious risk factor of fluke infection [17].

A strong association between liver fluke infection and CCA has been observed, and a mechanism has been proposed [18–21]. The pathway by which liver fluke infection leads to CCA is complex with multiple junctions that can be influenced by environmental exposures. It is hypothesized that the fluke itself resides in the bile ducts where it injures the lining and produces abnormalities such as advanced periductal fibrosis. This in turn activates the body's wound-repair response, which can lead to altered cell states and DNA damage [20].

In addition, substances that damage DNA may be produced by the body's inflammatory response to the infection [20–22]. Yet another source of cell modification and cancer risk includes substances directly secreted by the fluke. This storm of cellular modifications and DNA damage may lead to fixed genetic alterations that may contribute to cancer [18–21].

Thus, combined with other environmental factors, the liver flukes themselves as well as the processes they set into motion have ample time to cause the DNA damage that can ultimately lead to cancer [18–22]. Importantly, while liver fluke infection is strongly associated with CCA, it is not required for the disease to occur. Many cases have been documented in the absence of fluke infection [23], and in animal and human studies, liver fluke infection leads to CCA in only a subset of those infected [20, 24].

**Figure 1:**
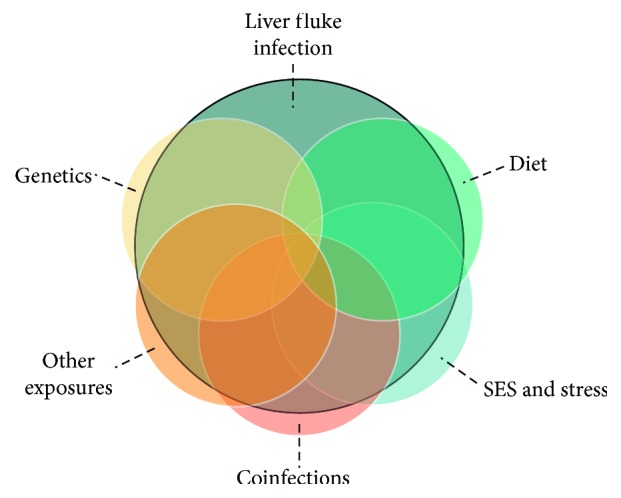
The complex factors involved in the pathway to CCA. Other environmental exposures may include nitrosamines, polycyclic aromatic hydrocarbons, arsenic, aflatoxin, and occupational exposures.

Whether or not an infection progresses to CCA is determined by the duration of the infection, intensity of the infection, genetics of the host, genetics of the liver flukes themselves, other viral infections, other diseases, diet, and other environmental exposures (Figure 1) [20, 25, 26]. Indeed, CCA has possibly the highest number of causative risk factors among all human malignancies [27] in addition to complex relationships between factors [26].

### 2.2. Diet and Other Environmental Exposures

In addition to the common practice of eating traditional dishes using raw or fermented fish that may carry liver flukes, a diet high in nitrosamines from salted fish fermented meats, sausages, and betel nuts is thought to contribute to the high incidence of CCA [21, 26, 28]. Animal studies suggest that nitrosamines not only contribute to cancer formation but in some cases may be required for carcinogenesis. For example, in hamsters, bile-duct wounding plus dietary nitrosamines resulted in development of cancerous lesions, while neither state alone resulted in lesions [29]. Similar findings were reported in other studies where hamsters infected with liver flukes for less than seven months developed cancerous lesions only when subcarcinogenic doses of nitrosamines were added to the diet [30].

Heavy cigarette smoking and alcohol consumption are other sources of nitrosamines and have been suspected to increase risk of CCA [12, 31]. The International Agency for Research on Cancer has also classified aflatoxins, which people can be exposed to through their diet, and arsenic, which they could be exposed to through drinking water, as carcinogens of the liver and bile duct with sufficient evidence and limited evidence, respectively [32].

Occupational exposures, including plutonium, aflatoxin, and vinyl chloride, have also been associated with liver cancer and CCA [32]. In Japan, occupational exposure to solvents has been associated with CCA [33]. Similarly, increased risk of CCA has been reported in workers exposed to asbestos, which researchers hypothesize involves an inflammatory pathway similar to liver fluke infection [34]. In Thailand, there has been some investigation into the relationship between occupational exposure to agricultural chemicals and CCA [35], but more information is needed about the potential role of these compounds, particularly in combination with other risk factors like liver fluke infection [26]. Other exposures that possibly have a relationship with CCA but require further study include polychlorinated biphenyls, dioxins, arsenic, and trichloroethylene [32, 36].

### 2.3. Genes and Genetic Variations

As with many cancers, individual differences play a role in the risk for CCA. Among CCA cases, only a subset has the long and intense inflammatory response to the flukes that contributes to cancer. For example, family history of cancer has been found to be related to CCA incidence [26]. It is further hypothesized that these people have a modified or abnormal regulation of inflammatory cytokine production in response to fluke infection that increases their risk of developing bile duct cancer, and that differences in inflammatory responses could be due to genetic variations [20]. At least one researcher has suggested that dietary and other exposures lead to CCA only in the presence of polymorphisms in DNA repair genes [37]; others are exploring the connection with variation in genes controlling metabolic functions, growth factors, and metabolism of xenobiotics [26]. Exploring these questions will lead to important discoveries. For example, in an early study, whole exome sequencing of CCA cases associated with *O. viverrini* infection identified mutations in 10 genes that were not previously implicated in cancer [8].

In addition, genetic variation among infectious vectors affects the course of complex diseases. For example, analysis of the genome and trancriptomes of *O. viverrini* and *C. sinensis* has found major differences between the two similar flukes, including differences in genes that alter proteins that play a large role in tissue migration, immune system evasion, and feeding—all stages that are crucial to the parasite's survival in the human host [19]. These genes and proteins represent possible targets for drugs or vaccines and need further study. Studying CCA will yield greater understanding of this process. The availability of the parasite's genome offers an opportunity for researchers to explore differences in infection rates among populations and the underlying causes, which can also inform comparative studies of understudied diseases [19, 38].

### 2.4. Coinfections and Comorbidities

Coinfections contribute to disease etiology and can exacerbate severity [39]. In the case of CCA, it is suggested that carcinogenic liver flukes have a symbiotic relationship with bacterium of the helicobacter species [39–44]. Infection with certain helicobacter species, *Helicobacter billis* and *Helicobacter hepaticus*, are thought to be involved in CCA based on studies in animal models [39] and in humans [43]. But results of efforts to detect these species in humans with bile duct cancer have been variable, though differences in methodology should be noted [39, 43]. To more fully understand the role of coinfections in complex diseases such as CCA, standardized diagnostic techniques such as PCR assays, immunohistochemistry antigens, and serological methods should be developed to promote early diagnosis and improve outcomes for patients [39]. Other coinfections whose relationships with CCA have been suggested but require further study include hepatitis C and hepatitis B [12,45–47], Epstein–Barr [48], and roundworms (*Ascaris lumbricoides*) [12].

Comorbidities are known to increase disease mortality and complicate intervention approaches [49]. In the case of CCA, some scientists have described the disease as a complication of chronic inflammation of the biliary system, or primary sclerosing cholangitis [14]. Indeed, an increased risk for CCA has been found among people who have a surgical biliary drainage procedure called anastomosis. Inflammation caused by the procedure is thought to be the inciting factor [12, 50]. Other comorbidities that may be related to CCA but require further study include inflammatory bowel disease, obesity, nonalcoholic liver disease, diabetes, and cirrhosis [17, 47, 48, 51, 52]. Studying comorbidities, such as those associated with CCA, can help researchers understand the influence of external exposures and other factors as well as shared underlying genes and pathways [49].

### 2.5. Social, Economic, and Psychosocial Factors

In Southeast Asia, more than 40 million people are infected with liver flukes. While liver flukes can be killed with praziquantel, people in Southeast Asia often get reinfected because of the persistent practice of eating raw fish, and due to socioeconomic factors that can delay or prevent access to treatment. These conditions make it common for people in this region to remain infected for long periods of time or even their whole lives, or to experience multiple cycles of infection followed by treatment [14, 20, 53]. A disproportionate number of CCA deaths in Thailand also occur in men, who often represent the major income earners for families, compounding the economic burden [20].

In addition, people in many rural communities in Southeast Asia are affected by a transition from farming to a more industrial economy, which may bring economic disparities and psychosocial stress [54–56]. The role of these factors in disease onset and progression has become an important area of research in recent years, with more attention focusing on the urgent need to understand how social and psychological factors are translated into disease risk [57–59]. For example, poverty exerts a chronic psychological stress on individuals and populations that has important implications for disease risk both by contributing to allostatic load and by predisposing populations to alcohol abuse and other risky behaviors [60].

The culturally important practice of food sharing, which promotes social cohesion and connection, has also been found to play a role in the risk for liver fluke infection, where households with more food sharing connections were more likely to have the infection [28]. These practices and other factors in human social ecology have been suggested as important considerations in understanding the transmission dynamics of liver fluke infection and other similar illnesses [28].

Other social and economic factors may be protective against CCA. For example, education level was found to have an inverse relationship with risk of CCA [26]. Other factors that did not have significant relationships, such as marital status and occupation, have been suggested to represent a range of factors that may have complex relationships with other CCA factors without being directly related to disease outcome [26].

Exploring these factors and how they contribute to or mitigate the risk of disease in the context of CCA may yield many important insights of relevance to other diseases, such as cardiovascular disease, immune suppression, respiratory diseases, and metabolic conditions [57, 58], where populations face economic or social stressors.

### 2.6. Considerations for Prevention and Intervention

Our current policies and infrastructure encourage data collection on few environmental exposures and neglect the unique needs of many regions and countries in the developing world for prevention and intervention strategies. These policies result in many missed opportunities for intervention along the pathway to noncommunicable disease. Understanding how to design policy and effective interventions for such complex conditions is crucial for stemming the tide of CCA and other noncommunicable diseases.

Like other multifactorial diseases that require a comprehensive approach for effective prevention [37], regional prevention for CCA will require interventions targeted at infections or exposures at both the national and local level. For example, there is evidence that liver flukes adapt and coevolve with their animal and possibly human hosts in their local region [25, 26]. In addition, it is possible that humans in certain regions may develop disease tolerance, as has been seen with other helminth infections [61–63]. Therefore, mass administration of praziquantel may not be advisable, especially since repeated cycles of treatment followed by reinfection is associated with development of cancer [26] and may increase the risk of flukes in a region becoming drug resistant [25].

An alternative is targeted treatment based on infection intensity, genetic background, culture, and diet [26, 28, 60, 64]. Understanding the importance of local perceptions, social networks, and other factors will inform interventions that target key disease risk factors while promoting important local customs and improving intervention outcomes [28, 64]. Moving towards a more holistic approach that integrates environmental contributions, evolution, biology, psychology, and social or behavioral dimensions will provide valuable insight towards addressing a variety of complex public health problems in addition to CCA [26, 60].

Early detection is another important part of prevention and intervention. In complex diseases that involve interplay between infection, inflammation, and environment, it is imperative to embrace advances in gene sequencing and expression to accelerate biomarker discovery to identify the early-stage biological processes that lead to cancer or other complex diseases. With CCA, a noninvasive, specific, and sensitive diagnostic marker is needed to increase early detection and treatment to improve survival rates [11, 65]. The stages between liver fluke infection and cancer are well characterized, but tests are needed to determine when an individual is in the midst of one of these stages so that intervention can begin. For instance, elevated concentrations of IL-6 in plasma have been shown to be a marker for the advanced fibrotic changes in the liver ducts that accompany bile duct cancer [20]. The Thai Ministry of Public Health has begun using plasma IL-6 screening, along with other tests, to detect early liver cancer in areas where infection with the *O. viverrini* liver fluke are common [20]. Advances in gene sequencing are revealing more about the biological activity involved in CCA and could possibly be used to diagnose infections with specific flukes [66]. Researchers also point to the need to make use of proteomics, or the large-scale study of proteins and their functions, to rapidly compare multiple pathways of protein expression in infected people who progress to cancer and those who do not [20].

Cancer and other complex, noncommunicable diseases have heterogeneous causes and mechanisms as well as highly variable geographic distribution. While efforts to prevent disease and control cancer must be individualized by location [67], exploring the many complex factors that contribute to CCA risk may help identify parallels that can be translated to other diseases and different populations.

## 3. Conclusions

Largely overlooked outside of the scientific community in Southeast Asia, CCA is a readily studied model that could improve our understanding of cancer [37] and potentially a large swath of other noncommunicable diseases. Many of the steps along the pathway from liver fluke infection to cancer are known, and a well-defined animal model of the disease is in place [20].

Many different cancers result from a scenario similar to CCA—an interaction between infectious agents, environmental exposures, and genetic susceptibility that increases allostatic load and can lead to disease [68]. While we currently lack many of the tools needed to get a full picture of the health impacts of complex environmental exposures [69], working to understand complex rare diseases like CCA provides an opportunity to develop such tools and improve our understanding of the interplay of multiple, heterogeneous factors in disease. It is time to focus more research attention on this long-neglected disease.
